# Heterozygous individuals with mild phenotype in late-onset glycogen storage disease type 2: a new cohort of patients?

**DOI:** 10.1186/1471-2474-14-S2-P12

**Published:** 2013-05-29

**Authors:** L Vercelli, E Vittonatto, S Grifoni, L Chiadò-Piat, E Rolle, M Spada, C Danesino, G Comi, T Mongini

**Affiliations:** 1 Centre for Neuromuscular Diseases, Department of Neuroscience, University of Turin, Turin, Italy

## Introduction

Late-onset glycogen storage disease type 2 (GSD2) is a genetic but heterogeneous disorder, which may present anywhere along a continuum of severity from an isolated hyperCKemia to a profound, generalized muscle weakness with pulmonary involvement. The gold standard for diagnosis is confirmation of low or absent levels of acid alpha-glucosidase (GAA) enzyme activity (usually in the range of 1-40% of normal levels), which is confirmed only in some cases by molecular analysis of the *GAA* gene. In the literature, heterozygous individuals are usually considered to be asymptomatic, although they can have reduced enzymatic activity. Since enzyme replacement therapy (ERT) became available in 2006, it has improved the prognosis for severe infantile-onset Pompe disease, as well as for late-onset forms by improving muscle/respiratory function and/or stabilizing clinical progression. Because the disease is now treatable, it is essential to understand which patients may benefit from ERT.

## Results

In the database of the Centre for Neuromuscular Diseases, we found 7 patients with only one mutated GAA allele: 2 female patients showed proximal weakness, pathologic muscular biopsy and reduced GAA enzyme activity in muscle tissue. These patients started ERT four years ago, and now there is no evidence of disease progression. The other 5 subjects (two are familiar cases, one father and his son) have mild signs of myopathy (i.e., slight scoliosis, proximal weakness, hyperlordosis, fatigability, recurrent episodes of respiratory deficit) or isolated hyperCKemia. Muscle biopsies showed non-specific signs, and reduced enzymatic activity (below 40%) was confirmed in all patients using at least two methods (dried blood spot and/or leukocytes and/or muscle homogenate).

## Conclusion

Heterozygotes with one GAA gene mutation are not always asymptomatic individuals; in our experience, they can develop a mild myopathy, have non-specific muscle biopsy results, and reduced GAA enzyme activity. We followed and monitored this cohort of patients (not receiving ERT) and found that they did develop clinical evidence of disease. In conclusion, mild symptomatic subjects and heterozygotes are an emerging group of patients. Further molecular investigations and follow-up are required to identify patients in this cohort that may benefit from ERT.

